# Phytocannabinoids CBD, CBG, and their Derivatives CBD-HQ and CBG-A Induced In Vitro Cytotoxicity in 2D and 3D Colon Cancer Cell Models

**DOI:** 10.3390/cimb46040227

**Published:** 2024-04-19

**Authors:** Dorota Bęben, Oliwia Siwiela, Anna Szyjka, Michał Graczyk, Daniel Rzepka, Ewa Barg, Helena Moreira

**Affiliations:** 1 Faculty of Pharmacy, Wroclaw Medical University, Borowska Street 211, 50-556 Wroclaw, Poland; dorota.biegas@student.umw.edu.pl (D.B.); oliwia.siwiela@student.umw.edu.pl (O.S.); 2 Department of Basic Medical Sciences and Immunology, Faculty of Pharmacy, Wroclaw Medical University, Borowska Street 211, 50-556 Wroclaw, Poland; anna.szyjka@umw.edu.pl (A.S.); helena.moreira@umw.edu.pl (H.M.); 3 Department of Palliative Care, Faculty of Health Sciences, Collegium Medicum in Bydgoszcz, Nicolaus Copernicus University, 87-100 Torun, Poland; michal.graczyk@cm.umk.pl; 4 Independent Researcher, 85-005 Bydgoszcz, Poland; daniel.rzepka7@gmail.com

**Keywords:** colon cancer, phytocannabinoids, *Cannabis sativa* L., cannabidiol, cannabigerol, cannabidiol hydroquinone, cannabigerolic acid, SW-620 cell line

## Abstract

Phytocannabinoids, compounds found in *Cannabis sativa* L., are used in oncology and palliative care to reduce the adverse reactions of standard therapies. Cancer patients use formulations of *Cannabis sativa* L. to manage the anxiety, pain, and nausea associated with cancer treatment, and there is growing evidence that some of them may exhibit anticancer properties. In this study, we tested the anticancer potential of selected cannabinoids CBD (cannabidiol) and its quinone derivative CBD-HQ (cannabidiol hydroquinone), CBG (cannabigerol) and its acid derivative CBG-A (cannabigerolic acid), as well as a combination of CBD+CBG on the colon cancer cell line SW-620. The MTT assay was used to determine the cannabinoids’ ability to induce colon cancer cell death. All cannabinoids were cytotoxic at the lowest concentration (3 μg/mL). The half maximal inhibitory concentration (IC50) ranged from 3.90 to 8.24 μg/mL, depending on the substance. Cytotoxicity was confirmed in a 3D spheroidal cell culture with calcein and propidium iodide staining. The amount of intracellular reactive oxygen species (ROS) was examined using a DCF-DA assay. CBG showed the lowest antioxidant activity of all the cannabinoids tested. The level of intracellular ROS decreased only by 0.7–18%. However, CBG-A induced the strongest reduction in ROS level by 31–39%. Our results suggest that cannabinoids represent an interesting research direction with great implementation potential. These preliminary results represent the beginning of research into the potential of these substances for anticancer treatment and underscore the potential for further research.

## 1. Introduction

The high incidence of cancer and costly anticancer therapies are not only a financial burden but, more importantly, carry a potential risk of harmful effects on the human body. Cancer mortality varies from country to country and is linked to several factors, such as prevention programs and the degree of public awareness. The search for substances with anticancer effects and effective therapies is prompting scientists to explore the potential of natural plant substances and the prospects of applying them to medicine. In this regard, there is growing interest in cannabinoids among researchers, healthcare professionals, and, above all, patients who are affected by this problem.

It is well known that cannabinoids have effects used in palliative care on some cancer-related symptoms (pain, nausea/vomiting, loss of appetite, increased muscle tension, anxiety, and depression). Clinical trials with small groups of patients and case reports indicate the benefits of medical cannabis, while clinical trials with larger groups of patients for the treatment of nausea and vomiting, pain, and cachexia have not demonstrated improvements compared to other available treatments [1,2].

In addition, evidence over the past several years confirms that these compounds can reduce tumor growth in mouse models of cancer. Cannabinoids have been shown to activate an endoplasmic reticulum stress-related pathway that leads to the stimulation of autophagy-mediated tumor cell death. In addition, cannabinoids inhibit tumor angiogenesis and reduce tumor cell migration. Mechanisms of resistance to the anticancer effects of cannabinoids have also begun to be investigated, as well as possible strategies for developing cannabinoid-based combination therapies to fight cancer [2,3,4].

Orally administered CBG has been shown to reduce colonic inflammation [5], and in another study, it was found to reduce tumor formation in an azoxymethane cell culture model of colorectal cancer, as well as reduce xenograft tumor growth [6].

Although there is a lack of clinical evidence to support the potential anticancer action of cannabis, preclinical research has shown its potential. Numerous stories shared online through social media platforms indicate that over 40% of cancer patients who use cannabis believe it will treat their cancer. Peer-reviewed publications have published case reports, but frequently these lack crucial clinical data to support anticancer claims [7].

Of the 207 preclinical articles reviewed, 107 (52%) were preclinical studies with original data. Findings revealed 77 case reports of patients who used cannabis to treat a variety of malignancies, including pancreatic, lung, prostate, gynecological, breast, and central nervous system cancers. Of the case reports, the review’s authors determined that 14% were strong, 5% were moderate, and the remaining 81% were weak. Pediatric patients were included in just 10% of the instances. A range of 10 to 800 mg of CBD per day were the most often reported doses for this anticancer cannabinoid. Six trials with THC dosages ranging from 4.8 to 7.5 mg were reported. Survival information for patients with recurrent glioblastoma multiforme was provided in two short studies [7].

Preclinical studies describing the anticancer potential of cannabis have led the public to believe that it can help treat cancer. Most of the available published data, including case reports, are of insufficient quality to support this claim. Cannabis can be used for symptomatic treatment in cancer patients, but outside of clinical trials, it should not be used for anticancer treatment instead of traditional evidence-based therapies. Thus, it is essential to plan future studies using cannabis and the phytocannabinoids it contains as anticancer agents in patients with advanced cancers. It is also necessary to identify the potential of individual cannabinoids and their dosage against different types of cancer.

Much is already known about the properties of CBD and CBG, but less about their derivatives. It should be noted that modification of the molecules, which takes place under conditions of improper storage of cannabis products, can have a significant impact on their activity. Phytocannabinoids can occur in acidic forms (e.g., CBG-A—cannabigerolic acid), and some of them, due to oxidation, can form a quinone structure (e.g., CBD-HQ—cannabidiol hydroquinone). Our study aimed to evaluate the anticancer potential of selected phytocannabinoids and their derivatives on a colorectal cancer cell line and to assess the effect of differences in molecular structures on antioxidant activity.

## 2. Materials and Methods

### 2.1. Cell Culture

The SW-620 colon cancer cells purchased from ATCC (Manassas, VA, USA) were cultured in DMEM/F-12 medium (Sigma Aldrich, Darmstadt, Germany) supplemented with 10% fetal bovine serum (FBS, Sigma Aldrich, Darmstadt, Germany) and concentrated gentamicin solution (Sigma Aldrich, Darmstadt, Germany). The cells were cultured under appropriate conditions (37 °C, 5% CO_2_). The medium was exchanged, and cells were passaged every 2–3 days, depending on confluence.

### 2.2. Compounds

**Figure 1:**
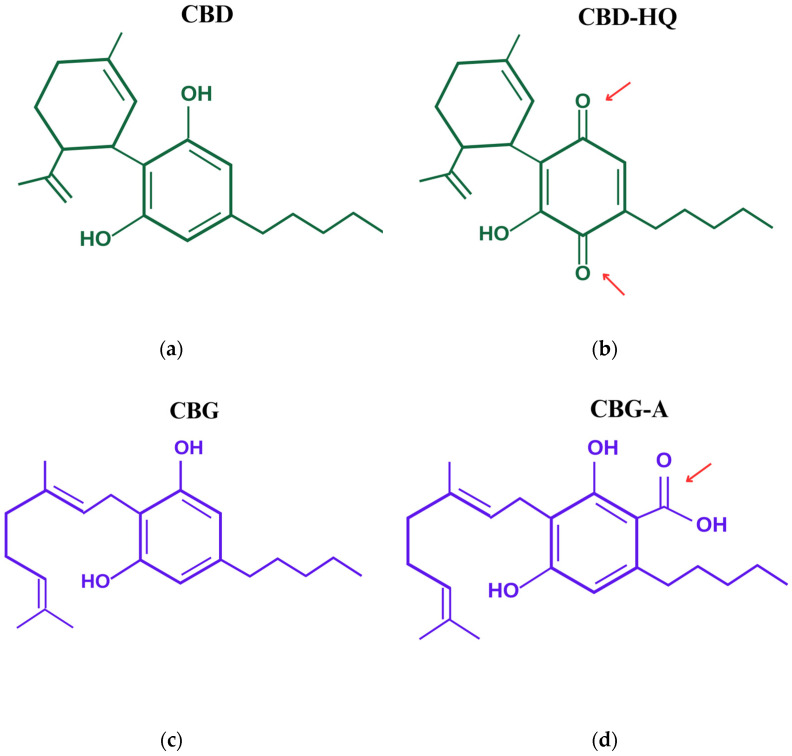
Structural formulas: (**a**) CBD, (**b**) CBD-HQ, (**c**) CBG, and (**d**) CBG-A The red arrows highlight the functional groups responsible for enhancing the antioxidant effect.

CBD (cannabidiol), CBD-HQ (cannabidiol hydroquinone), CBG (cannabigerol), and CBG-A (cannabigerolic acid) (Figure 1) were donated by the company Biomi-Farm. CBD (98%), CBG (98%), and CBG-A (96%) were isolated by double-recrystallizing *Cannabis sativa* L. distillate in alcohol. CBD-HQ (95%) was obtained by converting the CBD with KOH in alcohol. The company confirmed the quality of the test substances by gas chromatography-mass spectrometry (GC-MS). Stock solutions (SS) at a concentration of 1 mg/mL were prepared in dimethylsulfoxide (DMSO) (Sigma Aldrich, Darmstadt, Germany). Final concentrations of 1.5, 3, 6, 8, 10, and 12 μg/mL were obtained by diluting SS in DMEM/F-12 medium (Sigma Aldrich, Darmstadt, Germany). The concentration of DMSO in working solutions did not exceed 1.26%.

### 2.3. MTT Assay

2D cell culture was prepared in 96-well plates at a concentration of 30,000 cells/well. The cells were incubated for 24 h under optimal conditions (37 °C, 5% CO_2_) to adhere to the bottom. After this time, 200 μL of test compounds were added. The MTT assay was performed for single cannabinoids (CBD, CBG, CBD-HQ, and CBG-A) and a combination of CBD+CBG. Treated cells were incubated for 48 h. After this period, the contents of the wells were extracted. Then 50 μL of MTT salt (3-[4,5-dimethylthiazol-2-yl]-2,5-diphenyltetrazolium bromide) purchased from Sigma Aldrich (Darmstadt, Germany) in Minimal Essential Medium (MEM) (Sigma Aldrich, Darmstadt, Germany) at a concentration of 1 mg/mL was added. Plates were incubated at 37 °C with 5% CO_2_ for 2 h. The MTT solution was then extracted, and 100 μL of isopropanol was added. The plates were incubated in the dark at room temperature for 30 min. Reading was performed by spectrophotometry at wavelength 570 nm on a Perkin Elmer Wallac 1420 Victor 2 (PerkinElmer Life Sciences, Zaventem, Belgium). Experimental and control samples were compared to assess cell viability [8]. Results are presented as the percentage of viable cells relative to the control and the IC50 value (half maximal inhibitory concentration) with corresponding standard deviations. The experiment was performed three times, independently.

### 2.4. Spheroids

The plates used were 96-well plates with a U-shaped bottom and a less adherent surface. Cells were seeded onto a plate at a concentration of 3000 cells/well. The plates were centrifuged at 2000 rpm for 5 min, and then the prepared cells were left for 3 days in an incubator (37 °C, 5% CO_2_) to form a 3D structure [9]. On the fourth day, treatment was performed. After 48 h, 5 μL of calcein and 1 μL of propidium iodide (IP) from a Cellstain double staining kit (Sigma Aldrich, Darmstadt, Germany) were added to each well. Fluorescence intensity was measured using the Agilent BioTek Lionheart FX Automated Microscope (Agilent Technologies, Santa Clara, CA, USA) at excitation (about 501 nm) and emission (about 521 nm) for calcein and at excitation (about 488 nm) and emission (about 617 nm) for IP.

### 2.5. DCF-DA Assay

The level of intracellular reactive oxygen species (ROS) was measured using the DCF-DA (2′,7′-dichlorofluorescin diacetate) assay. 2D cell culture and the addition of substances proceeded as in the MTT assay. In addition to the negative control (medium without substance), a positive control (H_2_O_2_ 200 μM) was performed to evaluate the assay’s validity. After 48 h of incubation under standard conditions (37 °C, 5% CO_2_), cells were washed twice with phosphate-buffered saline (PBS) and stained with the fluorescent DCF-DA (Sigma Aldrich, Darmstadt, Germany) in a volume of 25 μL. The cells were then incubated (37 °C, 5% CO_2_) for 45 min. Fluorescence intensity was measured using the Varioskan LUX microplate reader (Biotek, Winooski, VT, USA) at excitation 485 nm and emission 535 nm wavelengths [8]. The results are displayed as a percentage relative to the negative control.

### 2.6. Statistical Analysis

All experiments were performed in triplicate. Statistical analysis and graphs were performed using GraphPad Prism 8.0.1 for Windows software (GraphPad Software, San Diego, CA, USA). Results were presented as mean ± standard deviation (SD). Differences between groups were examined using the Student’s *t*-test. Statistical significance for single substances was calculated relative to the control (culture without test substances), while the combination of CBD+CBG was measured relative to CBD and CBG alone. Statistical significance is shown as a *p*-value < 0.05. **** *p* ≤ 0.0001, *** *p* ≤ 0.001, ** *p* ≤ 0.01, * *p* ≤ 0.05.

## 3. Results

### 3.1. Study of the Capacity of Cannabinoids to Eliminate Colon Cancer Cells

**Figure 2:**
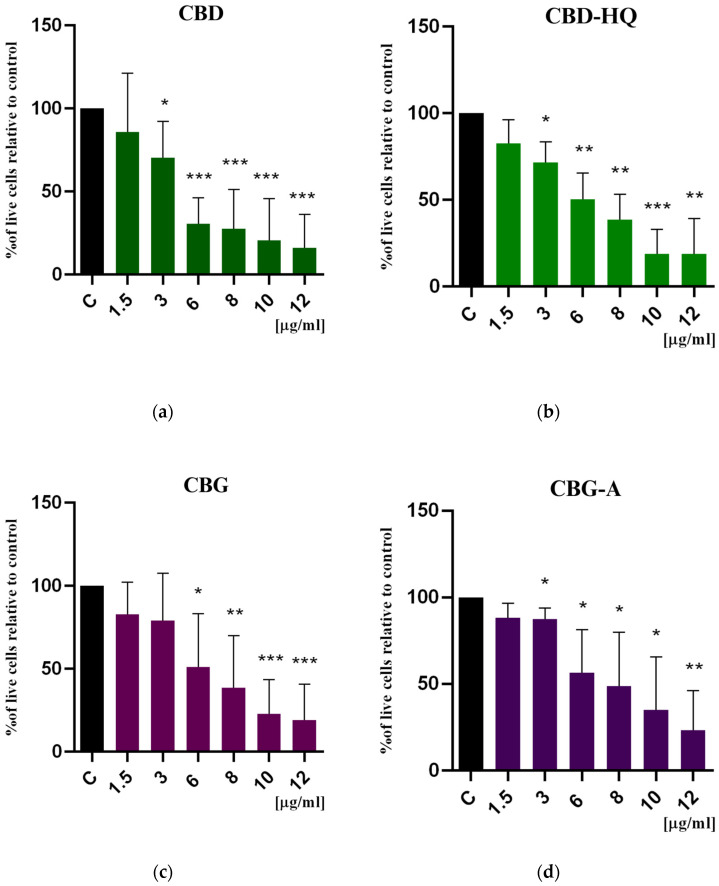
Cytotoxic effect of (**a**) CBD, (**b**) CBD-HQ, (**c**) CBG, and (**d**) CBG-A on colon cancer cells after 48 h of treatment. Results are presented as a percentage (%) of viable cells relative to the control (colon cancer cells incubated in the presence of the solvents) and are the mean ± SD of three independent experiments. The significance of the differences was determined by the Student’s *t*-test. *** *p* ≤ 0.001, ** *p* ≤ 0.01, * *p* ≤ 0.05 compared to the control group. Abbreviations: CBG—cannabigerol; CBG-A—cannabigerolic acid; CBD—cannabidiol; CBD-HQ—cannabidiol hydroquinone.

**Figure 3:**
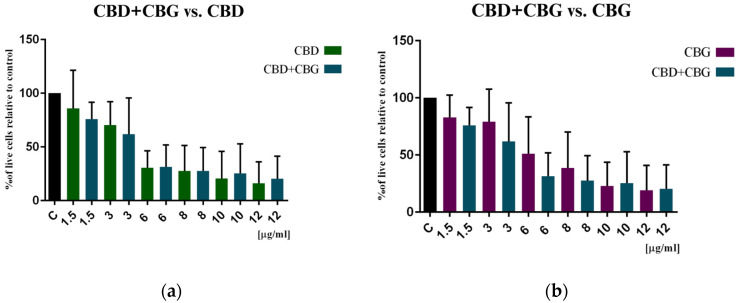
Cytotoxic effect of (**a**) CBD+CBG compared to CBD and (**b**) CBD+CBG compared to CBG on colon cancer cells after 48 h of treatment. Results are presented as a percentage (%) of viable cells relative to the control (colon cancer cells incubated in the presence of the solvents) and are the mean ± SD of three independent experiments. The significance of the differences was determined by the Student’s *t*-test compared to CBD and CBG alone, respectively. Abbreviations: CBG—cannabigerol; CBD—cannabidiol.

To determine the anti-tumor effect of cannabidiol, cannabidiol hydroquinone, cannabigerol, and cannabigerolic acid on colon cancer cells, an MTT assay was used. This assay is based on the activity of the cytoplasmic oxidoreductase enzyme as an indicator of cell viability. The results are presented in Figure 2 and Figure 3. The IC50 (half maximal inhibitory concentration) values are summarized in Table 1.

As shown in Figure 2, all tested compounds inhibit cancer cell viability in a dose-dependent manner, and the effect was statistically significant when compared to the controls (single substances) and when comparing CBG and CBD separately (combination) at almost all tested concentrations. Only at the lowest concentration (1.5 μg/mL), the cytotoxic effect did not reach statistical significance; however, colon cancer cell viability was affected by 24% for CBG-A and by 12–15% for the other cannabinoids.

The strongest cytotoxic effect on colon cancer cells was noted for CBD, with the lowest IC50 value compared to other cannabinoids. CBD, at concentrations of 6–12 μg/mL (19–38 μM/mL), decreased cancer cell viability by 63–73%. CBD-HQ, a quinone derivative of CBD, showed similar activity; however, its effect was weaker at concentrations of 6 and 8 μg/mL (18 and 24 μM/mL), where cell viability decreased by 71% and by 50%, respectively.

CBG also caused a significant reduction in colon cancer cell viability, comparable to the effect of CBD-HQ. The acid form of CBG, CBG-A, demonstrated the weakest cytotoxic effect compared to other cannabinoids, but it was still strong. CBG-A induced a reduction in cell viability by 65% (at 6 μg/mL = 17 μM/mL) to 77% (at 12 μg/mL = 33 μM/mL). The IC50 values for CBD-HQ, CBG, and CBG-A were comparable.

Furthermore, we tested whether the combination of CBD+CBG would exert a greater cytotoxic effect than both phytocannabinoids used separately. Only the concentrations of 1.5 and 3 μg/mL (5 and 9.5 μM/mL, respectively) of the CBD+CBG combination had a stronger effect compared to CBD alone or CBG alone. At concentrations of 6 and 8 μg/mL (19 and 25 μM/mL, respectively), the combination of both phytocannabinoids produced a greater cytotoxic effect than CBG alone but less than CBD alone. The highest tested concentrations of 10 and 12 μg/mL (32 and 38 μM/mL, respectively) had no stronger effect than both substances alone. Furthermore, the IC50 value calculated for the CBD+CBG combination is slightly lower than the IC50 value for CBD. As shown in Figure 3, the combination of the two phytocannabinoids did not show statistical significance compared to CBD and CBG alone, respectively.

### 3.2. Study of the Cannabinoid’s Effect on Spheroid Growth

Spheroid 3D cell culture allows for a better determination of the cytotoxic activity of the tested substances, as cells cultured in 3D models are more resistant to anticancer drugs. Staining the cells with calcein-AM (a marker of living cells) and PI (a marker of dead cells) allows a qualitative assessment of the viability of the cells forming the 3D culture.

**Figure 4:**
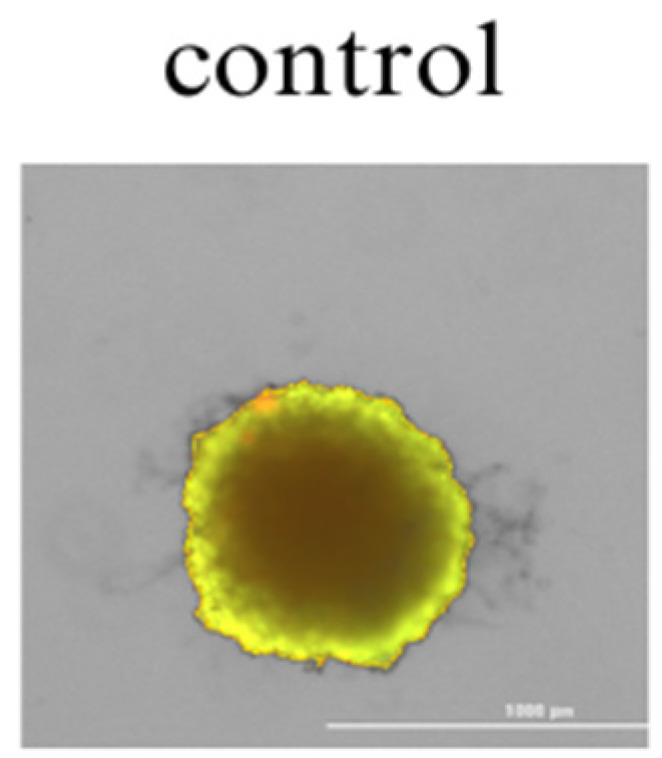
Spheroid 3D culture of colon cancer cells after 48 h of incubation with the solvent (DMEM F/12 medium) control culture (colon cancer cells incubated without the addition of cannabinoids). The scale bar is 1000 µm.

In the control sample (colon cancer cells incubated without the addition of cannabinoids), the 3D spheroidal culture was homogeneous and had a regular shape without any significant loss of structure (Figure 4). No dead cells were observed, indicating good cell viability in the 3D spheroid.

**Figure 5:**
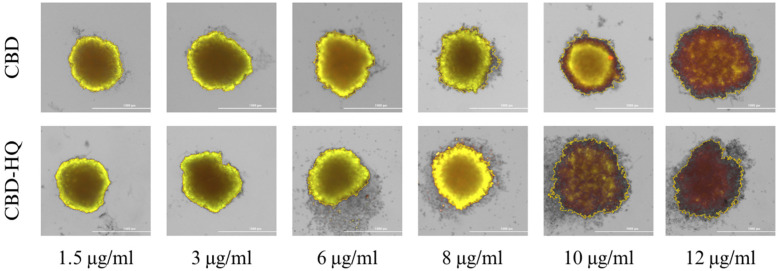
Spheroid 3D culture of colon cancer cells after 48 h of incubation with CBD and CBD-HQ. Abbreviations: CBD—cannabidiol; CBD-HQ—cannabidiol hydroquinone. The scale bar is 1000 µm.

**Figure 6:**
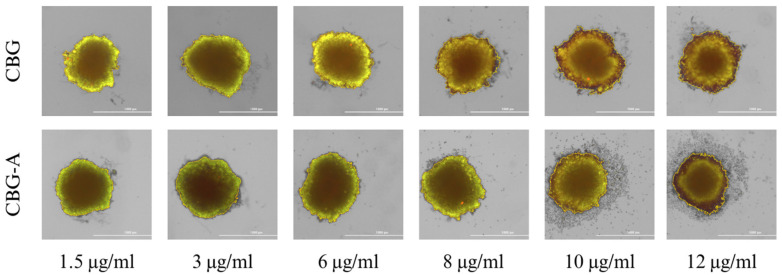
Spheroid 3D culture of colon cancer cells after 48 h of incubation with CBG and CBG-A. Abbreviations: CBG—cannabigerol; CBG-A—cannabigerolic acid. The scale bar is 1000 µm.

As shown in Figure 5 and Figure 6, all tested cannabinoids induced cancer cell death within the spheroid structure. This effect was especially seen when 6–12 μg/mL concentrations were applied to the cells. A less condensed and more dispersed structure of the spheroid can be observed. At the highest concentrations, the red color of propidium iodide is strongly visible, indicating significant necrosis inside the spheroid.

CBD-HQ demonstrated a stronger effect on colon cancer cells within the spheroid culture, indicating a stronger cytotoxic effect than CBD (Figure 5). CBD-HQ induced disruption of spheroid integration by detaching cells from the 3D culture, which was not observed with CBD.

Similar cell detachment from the spheroid structure was observed for CBG-A at the highest concentrations (Figure 6). However, CBG induced a stronger, more cytotoxic effect on the 3D cell culture, as evidenced by the intensity of the red color. The overall cytotoxic effects of CBG and CBG-A are weaker than those of CBD and CBD-HQ.

**Figure 7:**
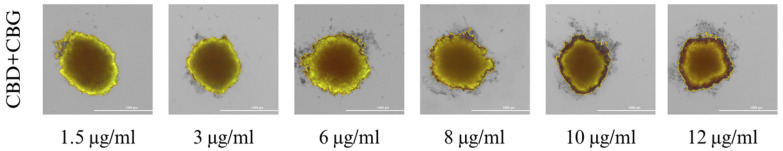
Spheroid culture after 48 h of incubation with a combination of CBD+CBG. Abbreviations: CBD—cannabidiol; CBG—cannabigerol. The scale bar is 1000 µm.

The combination of CBG+CBD produced a similar effect to CBG alone but was weaker than CBD alone (Figure 7).

### 3.3. Study of the Antioxidative Effect of Cannabinoids

**Figure 8:**
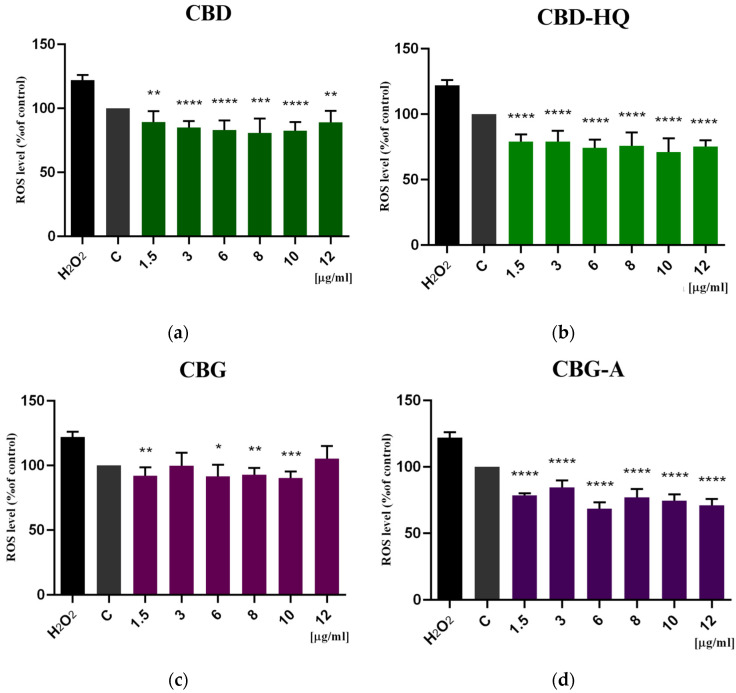
Induction of intracellular reactive oxygen species (ROS) by (**a**) CBD, (**b**) CBD-HQ, (**c**) CBG, and (**d**) CBG-A and H_2_O_2_ (200 µM) on colon cancer cells after 48 h of treatment. Results are presented as a percentage (%) of ROS quantity relative to the control (colon cancer cells incubated in the presence of the solvents) and are the mean ± SD of three independent experiments. The significance of the differences was determined by the Student’s *t*-test. **** *p* ≤ 0.0001, *** *p* ≤ 0.001, ** *p* ≤ 0.01, * *p* ≤ 0.05 compared to the control group. Abbreviations: CBG—cannabigerol; CBG-A—cannabigerolic acid; CBD—cannabidiol; CBD-HQ—cannabidiol hydroquinone.

**Figure 9:**
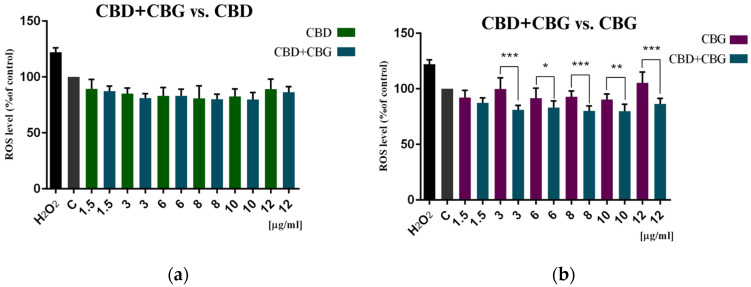
Induction of intracellular reactive oxygen species (ROS) by (**a**) CBD+CBG compared to CBD and (**b**) CBD+CBG compared to CBG and H_2_O_2_ (200 µM) on colon cancer cells after 48 h of treatment. Results are presented as a percentage (%) of ROS quantity relative to the control (colon cancer cells incubated in the presence of the solvents) and are the mean ± SD of three independent experiments. The significance of the differences was determined by the Student’s *t*-test. *** *p* ≤ 0.001, ** *p* ≤ 0.01, * *p* ≤ 0.05 compared to CBG alone and CBD alone. Abbreviations: CBG—cannabigerol; CBD—cannabidiol.

The influence of cannabinoids on the level of intracellular reactive oxygen species (ROS) in colon cancer cells was studied using the DCF-DA assay. The results are shown in Figure 8 and Figure 9. CBD caused a 17–26% decrease in the amount of intracellular ROS compared to the control cells. CBD-HQ exerted a greater antioxidant effect than CBD, resulting in a 20–34% reduction in ROS. CBG showed the lowest antioxidant activity of all the cannabinoids tested. The level of intracellular ROS decreased only by 0.7–18% compared to the control. In contrast, CBG-A induced the strongest reduction in ROS level by 31–39%.

The combination of CBD+CBG slightly improved the antioxidant effect of CBD and CBG. The ROS level decreased by 19–28% compared to the control cells. Compared to CBD alone, the combination of the two phytocannabinoids induced a lower amount of intracellular ROS by 0% to 5%. In contrast, compared to CBG alone, the amount of ROS released was lower by 4% to 30%. As shown in Figure 9, the difference was statistically significant only when compared to CBG.

## 4. Discussion

Colorectal cancer is of concern not only because of its incidence but also because of its high potential to metastasize [10]. By 2030, the incidence of colorectal cancer in the 20- to 34-year-old age group is estimated to increase by as much as 90%. Therefore, improving the treatment of this cancer is of urgent importance [11]. Standard therapies such as surgery, chemotherapy, and radiation therapy can be effective but are associated with serious side effects that affect patients’ quality of life. Removal of a cancerous lesion during surgery is an invasive procedure and carries long-term consequences, such as fecal incontinence and constipation. This represents long-term discomfort for the patient and makes it difficult to function in society (including working) [12]. Adjuvant therapy is often used after the removal of the cancerous lesion. Its implementation reduces the risk of death by only 3–5% in stage II colorectal cancer and by 10–15% in stage III. Moreover, not every patient can benefit from this type of cancer therapy. About 3–5% of patients have a deficiency in the enzyme that breaks down the administered drug, which can result in a lack of response to the drug and possible death after its administration. Therefore, before starting adjuvant chemotherapy, patients should undergo genetic testing to determine the enzyme’s ability to degrade the adjuvant, which involves additional costs [13]. It takes time to diagnose a patient, which is why the whole world faces the problem of delaying treatment. Cancerous lesions are characterized by rapid, exponential growth. Delaying the start of therapy by as little as four weeks significantly reduces a patient’s chance of survival [14]. In addition, diagnosing and treating colorectal cancer can be very expensive. The lack of effective and safe methods of treating colorectal cancer creates an urgent need to search for new therapeutic agents.

Medicinal plants are important sources of anticancer compounds. One of the compounds that have been identified and extracted from plants is camptothecin, found in *Camptotheca acuminata*, or paclitaxel, isolated from *Taxus brevifolia* [15,16]. Clinical studies also provide evidence of the anticancer effects of curcumin [17]. These substances are often structurally very complicated, so they can interact with multiple molecular pathways and can be used in adjuvant or chemopreventive therapy.

Recently, growing interest in medical research has focused on the plant *Cannabis sativa* L. This plant is rich in various phytochemicals with high therapeutic potential, including terpenes, flavonoids, stilbenes, lignans, alkaloids, and phytocannabinoids [18]. Phytocannabinoids are a structurally diverse class of chemical constituents of the plant, with over 100 different phytocannabinoids identified to date. The best known are 9Δ-THC (tetrahydrocannabinol), the main psychoactive component, and CBD (cannabidiol). A phytocannabinoid that is quickly gaining interest due to its high therapeutic potential is CBG (cannabigerol) [19]. Both CBD and CBG are devoid of psychoactive effects. In addition to plant phytocannabinoids, there are known endogenous cannabinoids produced in the body (e.g., anandamide) and synthetic cannabinoids produced as a result of chemical syntheses. All interact with receptors of the endocannabinoid system (ECS), a signaling pathway that regulates several physiological and pathological states in the body [20]. The effects of cannabinoids are primarily mediated by cannabinoid type 1 (CB1) and type 2 (CB2) G-protein-coupled receptors (GPCRs). Binding to the receptor induces a wide variety of intracellular actions, such as inhibition of adenylyl cyclase and some voltage-sensitive calcium channels, stimulation of mitogen-activated protein kinases (MAP kinases) and inwardly rectifying potassium channels (GIRK), and recruitment of beta-arrestin [21]. This has a variety of consequences for cellular physiology, including synaptic function, gene transcription, and cell motility [22].

CB1 and CB2 receptors are located in cancer cells of triple-negative breast cancer and high-grade glioma, but also in pancreatic duct epithelial carcinoma cells and colon cancer cells, including those of the SW-620 cell line [23,24,25]. The presence of ECS receptors in the SW-620 cell line has motivated us to include this particular colon cancer cell line in our research. The multidirectional effects of ECS render this system a potential therapeutic target for many disorders. Moreover, it may influence the regulation of chemotherapy-induced nausea and vomiting and cancer-related cachexia, which highlights its importance in cancer therapy [26]. Phytocannabinoids exhibit a wide range of activity due to their affinity for G-protein-coupled receptors, vanilloid channels, and peroxisome proliferator-activated receptor gamma (PPARγ). As a result, they can reduce inflammation, exert antimicrobial, antifungal, and antioxidant effects, or lower glycemia [27,28]. In cancer patients, medical marijuana (MC) has been reported to reduce pain, improve appetite and sleep, and reduce night sweats [29]. Patients undergoing chemotherapy and radiation therapy are very fatigued by both the drugs and the disease process itself. Current therapies debilitate the body, often leading to reduced immunological function, chronic inflammation, and chronic fatigue. In addition to the general anti-inflammatory properties of phytocannabinoids, they appear to inhibit the penetration of the SARS-CoV-2 virus into human cells. It was reported that they are allosteric and orthosteric ligands with micromolar affinity for the spike protein. They show effectiveness against both alpha and beta variants of coronavirus [30].

The most common form of phytocannabinoid intake is aspiration of the smoke produced by smoking MC or using cannabis oils sublingually [31]. Formulations in the form of vape-pens or cartridges for e-cigarettes are now also becoming more common. Among those who vaporize cannabis, as many as 59.1% reported vaporizing cannabis oil or liquids, and 34.0% reported vaporizing cannabis concentrates [32]. The high temperatures accompanying this process cause changes in the chemical structures of the substances present in the extract. Also, the method of storage can have a significant impact on their effectiveness in pharmacotherapy. When exposed to light and access to air, CBD is transformed into CBD-HQ. Oxidation of CBD results in the formation of a hydroquinone group, which can potentially alter the therapeutic effect of CBD [33]. Decarboxylation of CBG-A to CBG also occurs under these conditions. Not all cannabis oils have acidic forms in their composition, as this depends on the method of preparation. The use of steam distillation results in the apparent decarboxylation of acids. Oils obtained by this method are characterized by a minor quantity of cannabinoid acids [34]. If CO₂ extraction, oil extraction, cold pressing, or solvent extraction (e.g., with ethanol) is used, the acid forms will be found in higher concentrations, and heating the product will change the qualitative and quantitative composition by decarboxylation, as mentioned above [35].

Here, we have studied the potential of three phytocannabinoids: cannabidiol (CBD), cannabigerol (CBG), its acid precursor (CBG-A), and one synthetic cannabinoid, a quinone derivative of CBD (CBD-HQ), to inhibit colon cancer cells using the SW-620 cell line. CBD, as well as CBG and CBG-A, are presumed to inhibit colon cancer growth by inducing apoptosis and inhibiting angiogenesis and the cell cycle [36]. We have demonstrated very strong cytotoxic properties of CBD, CBD-HQ, CBG, and CBG-A on cancer cells. This effect was statistically significant (*p* < 0.05) compared to the control. The IC50 values for all cannabinoids were very low, with a maximum of 8.24 μg/mL.

Phytocannabinoids can interact with each other, leading to synergistic effects. The combination of phytocannabinoids can significantly increase their cytotoxicity toward cancer cells [37]. To verify this hypothesis, the combination of CBD+CBG was studied. Our cytotoxicity studies (MTT, spheroids) showed no significant improvement in activity compared to CBD and CBG separately. The statistically significant difference was observed only in the DCF-DA assay compared to CBG but not to CBD.

CBD-HQ is a much less studied cannabinoid. Currently, there is speculation about its use in qualitative analysis because, as an oxidized CBD derivative, it may indicate improper storage of CBD products. Further studies are needed to determine its pharmacological properties as well as how potentially improper storage of CBD products could affect its biological effects [33]. It has been postulated that CBD-HQ may affect the metabolism of drugs metabolized by cytochrome P-450 by inhibiting the microsomal enzymes of this cytochrome [38]. A mouse study showed that the microsomal metabolism of CBD to CBD-HQ in hepatocytes is paralleled by the formation of ROS, which may be responsible for hepatotoxicity [39]. In our study, both CBD and CBD-HQ did not increase the level of intracellular ROS in colon cancer cells. CBD-HQ caused a reduction in ROS release by 34%. However, given the isolated reports of hepatotoxicity of both cannabinoids, the MC therapy of patients with liver failure should be carried out with caution. The anticancer activity of CBD-HQ might be related to the inhibition of the enzyme responsible for replication, transcription, and regulation of DNA chromatin topology. No inhibition of topoisomerase IIα and β activity was observed by CBD [40]. The synthetic analog of CBD-HQ, the cannabinoid HU-331, was shown to have the ability to inhibit the growth of prostate and pancreatic cancer cells [41]. In our study, we have confirmed that CBD-HQ inhibits the growth of cancer cells, based on the example of colon cancer cells. A concentration of 8 μg/mL inhibits the growth of 50% of the cell population under study. The anti-tumor effect was also confirmed in 3D cell culture. We showed greater anti-tumor efficacy of CBD in 2D culture and greater efficacy of CBD-HQ in 3D culture, which would suggest a better anticancer effect of CBD-HQ in in vivo conditions.

In addition, we have observed that modification of the chemical structure (Figure 1) affects the antioxidant potential. The presence of certain functional groups may influence the ability of the cannabinoids to reduce the release of ROS by cancer cells. Considering both CBD and CBD-HQ, the hydroquinone structure of CBD-HQ allows the neutralization of ROS by donating hydrogen bonds. However, it should be noted that the process of converting CBD to CBD-HQ in the body may be associated with an increased release of ROS [42]. CBD-HQ, as well as CBG-A, are oxidized species that can be reduced and thus act as an oxidant. Both cannabinoids may participate in redox reactions and readily donate electrons, thereby stabilizing ROS. CBD is the more stable form, while CBD-HQ is a more reactive one. This may also affect the ability of both chemicals to respond to excess ROS secreted by cells. The antioxidant properties are affected by pH and the presence of other substances. Thus, the observed antioxidant effects may be quite different when administering a mixture of cannabinoids or even when administering isolates to a patient. It is worth noting that our study did not show a relationship between antioxidant efficacy and cytotoxicity against cancer cells. None of the cannabinoids tested showed pro-oxidant activity, indicating that their mechanism of anticancer action is not mediated by oxidative stress.

In conclusion, here we demonstrated the strong anticancer effects of CBD and CBG and their derivatives on colon cancer cells. Moreover, all cannabinoids showed slight antioxidant properties. The chemical structure is key to understanding its mechanism of molecular action. Substances of natural origin often have a multidirectional mechanism of action by affecting multiple cellular pathways. Such properties can have both positive and negative effects.

Like almost every pharmacologically active substance, cannabinoids have some adverse effects. Smoking marijuana can lead to inflammatory cell infiltration, which results in pneumonia. It can also affect the cardiovascular system by inducing transient ischemic attacks and arteritis. Severe adverse events such as stroke, seizures, myocardial infarction, rhabdomyolysis, acute kidney injury, and psychosis have also been reported [43].

Knowledge of the activity of individual components of *Cannabis sativa* L. will improve the safety of the therapy. Understanding possible structural modifications of phytocannabinoids during storage and use of formulations would allow for a more accurate prediction of therapeutic efficacy. Moreover, it seems important to adjust the quantitative and qualitative composition of the preparations used depending on the form of the drug. There is no doubt that phytocannabinoids represent an interesting research direction with great potential for implementation. To confirm the potential of cannabinoids in oncology in the next phase, it would be necessary to look at the molecular mechanisms of action in different types of cancer. It would also be worthwhile to consider the safety of the test substances against healthy cells and, in the future, also include in vivo studies. Interactions between cannabinoids and other components of cannabis, including terpenes, should be further studied.

## Figures and Tables

**Table 1:** Half maximal inhibitory concentration (IC50) of tested cannabinoids for the cytotoxic effect on colon cancer cells.

	CBD	CBD-HQ	CBG	CBG-A	CBD+CBG
IC50 [μg/mL]	4.13	8.00	7.64	8.24	3.9
IC50 [μM/mL]	13.13	24.35	24.14	22.86	12.36

## Data Availability

Data are contained within the article.
